# On the Role of Additional [4Fe-4S] Clusters with a Free Coordination Site in Radical-SAM Enzymes

**DOI:** 10.3389/fchem.2017.00017

**Published:** 2017-03-16

**Authors:** Etienne Mulliez, Victor Duarte, Simon Arragain, Marc Fontecave, Mohamed Atta

**Affiliations:** ^1^Biosciences and Biotechnology Institute of Grenoble, Laboratoire de Chimie et Biologie des Métaux, UMR 5249 CEA-Centre National de la Recherche Scientifique-UGAGrenoble, France; ^2^Laboratoire de Chimie des Processus Biologiques, UMR 8229, Collége de France-Centre National de la Recherche Scientifique-Université P. et M. CurieParis, France

**Keywords:** radical-SAM, iron-sulfur clusters, macromolecule modifications, metalloenzymes, C-H bound activation

## Abstract

The canonical CysXXXCysXXCys motif is the hallmark of the Radical-SAM superfamily. This motif is responsible for the ligation of a [4Fe-4S] cluster containing a free coordination site available for SAM binding. The five enzymes MoaA, TYW1, MiaB, RimO and LipA contain in addition a second [4Fe-4S] cluster itself bound to three other cysteines and thus also displaying a potentially free coordination site. This review article summarizes recent important achievements obtained on these five enzymes with the main focus to delineate the role of this additional [4Fe-4S] cluster in catalysis.

## Introduction

The amazing development of the Radical *S*-Adenosylmethionine (RS) enzymes is unique in the history of modern enzymology and covers three distinct periods. The early days of research (1970–1990) constitute the first period in which only three enzymes were concerned, namely: the lysine 2,3-aminomutase and both activases of pyruvate formate–lyase and class III ribonucleotide reductase (Fontecave et al., 2002; Frey et al., 2008; Shisler and Broderick, 2014). At that time the proposed reaction mechanisms for these three enzymes featuring unusual radical chemistry were considered as chemical curiosities, reminiscent of the chemistry at work in cobalamin-dependent enzymes (Frey and Magnusson, 2003; Marsh et al., 2010). Indeed, the reaction mechanism for the C-H bonds activation was then, and still is now, viewed as one of the most chemically demanding reactions in enzymology (Booker, 2009). It was then thought to be most often linked to those metalloenzymes using dioxygen as to create an oxidant strong enough to cleave C–H bonds (Lippard, 2005). The second period, from 1991 to 2001, was a key step in this development. During this time two new enzymes, biotin synthase and lipoyl synthase, were purified and investigated by biochemical and spectroscopic methods and found to share common features with the first three ones (Sanyal et al., 1994; Miller et al., 2000). In particular, their enzymatic activities were completely dependent on the simultaneous presence of two cofactors, a [4Fe-4S]^2+/1+^ cluster and S-Adenosyl-L-methionine (SAM). A common and essential CysX_3_CysX_2_Cys motif was identified within the five enzymes and shown to provide three of the four ligands to a [4Fe-4S]^2+/1+^ center, called RS cluster. The fourth coordination site was soon after proven to bind SAM via its α-amino-carboxylate moiety in a bidentate fashion (Walsby et al., 2002). When reduced, the [4Fe-4S]-SAM adduct leads to the reductive cleavage of the adenosyl sulfonium bond of SAM and generates a 5′-deoxyadenosyl radical (5′- Ado^•^), a very strong oxidant able to activate most of C-H bonds (Fontecave et al., 2001; Wang and Frey, 2007). These informations together with the spectacular soaring of genome sequencing allowed for a bioinformatic screening which promoted this marginal group of individuals to an important superfamily, appropriately denoted “Radical-SAM” (RS) enzymes. Since then, advanced sequence profiling methods have demonstrated that over 600 putative proteins involved in diverse cellular processes shared significant sequence similarities (Sofia et al., 2001). The third period, from 2002 until now, is witnessing the rapid growth of this superfamily of enzymes and the enhanced understanding of individual family members. In fact, since 2002, a large community of aficionados of this superfamily of enzyme is being constituted that thrives for exploring many biosynthetic pathways dependent on these enzymes. Thus, the discovery of the RS superfamily resulted in the revitalization in studying radical-dependent enzymatic reactions.

An extensive survey of the literature indicates that more than 50 structures of RS enzymes have been reported. One of the remarkable features, which up to now does not suffer any exception is that all of these enzymes adopt an invariant fold (Vey and Drennan, 2011). This fold most often consists in a partial (β/α)_6_ triose-phosphate isomerase (TIM) barrel although some enzymes exhibit a full (β/α)_8_ version of it (Vey and Drennan, 2011). In some occasions N- and C-terminal extensions appended to the Radical-SAM domain have been reported and shown to house other needed cofactors and/or to be involved in substrate recognition (Lanz and Booker, 2015). In this special issue of Frontiers in Chemistry we review the current state of knowledge on a five-member subgroup of the RS superfamily which displays, in addition to the RS cluster, a second [4Fe-4S] cluster also bound to the polypeptide chain by only three cysteine amino acid residues. These enzymes are the following: -MoaA enzyme which is involved in the first step in the conversion of guanosine-5′-triphosphate (5′-GTP) to Moco cofactor, -tRNA-4-demethylwyosine synthase, TYW1, that catalyzes the second step in the wybutosine (yW) biosynthesis occurring in the tRNA modification event, -MiaB and RimO defined as methylthiotransferases (MTTases) and discussed in the same section. Both enzymes catalyze the same chemical reaction, namely, the transfer of a methylthio group on a specific adenine of several tRNAs for MiaB and on a specific aspartate residue in the ribosomal protein S12 (RPS_12_) for RimO and finally LipA which inserts two sulfur atoms in the octanoyl chain of several important 2-oxoacid dehydrogenases as well as of the glycine cleavage system to afford the essential growth factor lipoate.

We will give an overview of important achievements obtained on these five enzymes with the aim to delineate the role of the additional [4Fe-4S] cluster in each enzyme and the evidences that sustain its proposed function. We will pay a particular attention to the case of the MTTases (MiaB and RimO) and as a supplement to the excellent reviews that have been already published we will describe how the hypotheses on the role of the additional [4Fe-4S] cluster have emerged and how these were experimentally substantiated in our laboratory (Forouhar et al., 2013). However, it is worth noting that a number of other RS enzymes with additional [4Fe-4S] cluster(s) have been reported and for most of them the role(s) of the additional cluster(s) remain to be established.

## MoaA enzyme: molybdenum cofactor (Moco) biosynthesis

**Figure 1:**
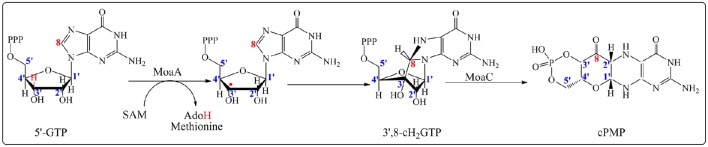
**Formation of cPMP catalyzed by MoaA and MoaC enzymes in the first step on the Moco biosynthesis**. MoaA catalyzes the abstraction of a hydrogen atom at 3′ position (in red) leading to the formation of 3′,8-cH_2_GTP intermediate. MoaC is involved in the cPMP formation. The carbons of the ribose moiety and guanosine base as determined by isotope labeling experiments are in blue and red respectively.

Molybdenum cofactor (Moco) is essential for many enzymes that catalyze diverse key reactions in the global metabolism of carbon, nitrogen, sulfur and is required for all kingdoms of life (Hille, 1996; Schwarz and Mendel, 2006). Unlike many other cofactors, Moco cannot be taken up as a nutrient, and thus it requires *de novo* biosynthesis (Leimkuhler et al., 2011; Mendel and Schwarz, 2011). The Moco cofactor biosynthetic pathway is a five-step process involving RS-based chemistry (Leimkuhler et al., 2011). The first step in the biosynthetic sequence is the conversion of guanosine-5′-triphosphate (5′-GTP) to a cyclic pyranopterin monophosphate (cPMP), known as precursor Z, and is catalyzed by the RS enzyme MoaA and the accessory protein MoaC (Hanzelmann and Schindelin, 2004, 2006). For a long time, the exact contribution of each protein in this step was unclear and the prevalent view was that MoaA was the major key player in the transformation of the 5′-GTP into cPMP intermediate with no catalytic role for MoaC. Recently, elegant functional and structural studies established that both enzymes MoaA and MoaC act synergetically to catalyze this amazing rearrangement. Now, the current view is that the RS MoaA enzyme catalyzes the transformation of 5′-GTP into 3′,8cH_2_GTP which is the true substrate of MoaC for cPMP formation (Hover et al., 2013, 2015a,b; Figure 1).

**Figure 2:**
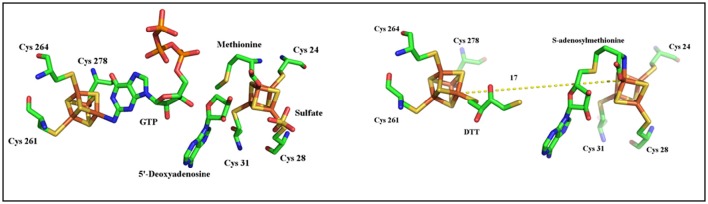
**Structures of MoaA in complex with 5′-GTP (left)** and DTT **(right)**.

As described above, MoaA belongs to a sub-group of the Radical-SAM superfamily which contains two [4Fe-4S] clusters essential for activity. Historically, it is considered as the prototype of this subclass. The N-terminal half of the protein contains the canonical CysX_3_CysX_2_Cys motif that binds the RS [4Fe-4S]^2+/1+^ cluster, in which the fourth, unique iron, is utilized to bind SAM (Hanzelmann and Schindelin, 2004, 2006; Lees et al., 2009). The additional [4Fe-4S]^2+/1+^ cluster is located at the C-terminal part of the enzyme and is also ligated to the polypeptide by three cysteines clustered in a CysX_2_CysX_13_Cys motif leaving the fourth, unique iron, with a free coordination site (Hanzelmann and Schindelin, 2004, 2006; Lees et al., 2009). The available structures showed that the two [4Fe-4S]^2+/1+^ clusters are 17 Å apart (Hanzelmann and Schindelin, 2004, 2006). Both crystallographic and ENDOR studies have shown that the additional [4Fe-4S]^2+/1+^ binds 5′-GTP substrate in a very unusual coordination involving the N_1_ purine nitrogen and the N_2_ exocyclic amino group (Hanzelmann and Schindelin, 2004, 2006; Lees et al., 2009). In the absence of 5′-GTP, this cluster was shown to bind a dithiothreitol (DTT) molecule (Figure 2; Hanzelmann and Schindelin, 2004).

The first step in the MoaA-catalyzed reaction (Figure 1) conforms to the unifying step in the RS enzymology resulting in the production of 5′- Ado^•^ that abstracts a hydrogen atom from the substrate. Note that one exception to this rule has been recently reported in the case of MqnE where the 5′-Ado^•^ radical adds to the enol ether double bond of 3-[(1-carboxyvinyl)oxy]benzoic acid (Mahanta et al., 2013).

As shown in Figure 1, activation of 5′-GTP starts with abstraction of a hydrogen atom from the C_3′_ of 5′-GTP to give a radical intermediate (Mehta et al., 2013a,b). The latter is then proposed to add to C_8_ of the purine with concomitant electron transfer from the purine bound to the additional [4Fe-4S] cluster. This radical addition reaction yields 3′,8cH_2_GTP which has been characterized (Hover et al., 2013) and shown to be processed in a complex rearrangement reaction to cPMP product by the action of MoaC (Hover et al., 2013, 2015a,b). In the reaction of MoaA, the role of the additional [4Fe-4S] cluster is critical since the binding the 5′-GTP allows for its positioning close to the produced 5′- Ado^•^ radical appropriate for direct H atom abstraction. Moreover, the binding mode of 5′-GTP to the unique iron (Figure 2) has two consequences. First, under physiological conditions, it allows the enzyme to discriminate the 5′-GTP substrate from the more abundant 5′-ATP analog and second, it facilitates the keto-enol tautomerization of the N_1_-O_6_ moiety that is thought to modulate the purine reactivity (Hanzelmann and Schindelin, 2006; Lees et al., 2009). Thus MoaA was the first RS enzyme with two [4Fe−4S] clusters for which the role of the additional [4Fe-4S] cluster was shown to promote activation of the substrate in a way most similar to that of the [4Fe-4S] cluster of aconitase (Kent et al., 1985). The ability of the additional cluster of MoaA to bind and activate its substrate constitutes a central theme in the properties of the enzymes of this subgroup as will be illustrated below.

## TYW1 enzyme: 4-demethylwyosine biosynthesis

**Figure 3:**
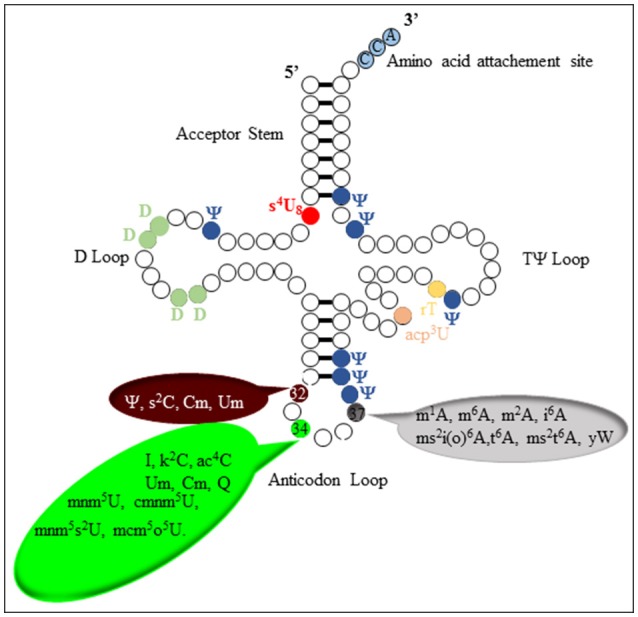
**Schematic representation of tRNA with some known modifications and their positions**.

To date more than 100 modified nucleosides have been structurally characterized (http://modomics.genesilico.pl/). They are found across the three kingdoms of life and many of them exhibit extremely deep evolutionary conservation (Grosjean, 2009). Some of the modifications are generated by relatively simple biosynthetic reactions involving methylation, thiolation, pseudouridylation, or dihydrouridine formation, and found in all regions of most tRNAs (Agris et al., 2007). On the contrary, some specific regions, the Anticodon Stem Loop (ASL) region (Figure 3) in particular, display more complex modifications called “hypermodifications,” which are proposed to mainly contribute to the stabilization of the codon-anticodon pair, thereby maintaining the translational fidelity with high efficiency (Agris et al., 2007).

**Figure 4:**
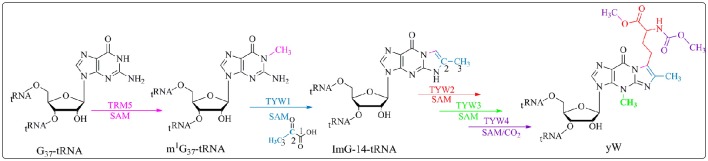
**Biosynthetic pathway of yW**. Trm5 methylates G_37_-tRNA to produce m^1^G_37_-tRNA; TYW1, a RS enzyme, catalyzes the formation of 4-demethylwyosine (imG-14) using pyruvate as a co-substrate. yW is produced upon subsequent reactions with TYW2, TYW3 and TYW4.

The distinctive chemistry of the biosynthetic pathway leading to these hyper-modifications requires several challenging enzymatic reactions. One of the most chemically intricate modification that occurs at position 37 of tRNA_Phe_ in eukaroytes and archaea is the formation of the wybutosine base (yW) which contains a fluorescent tricyclic fused aromatic base derived from a genetically encoded guanosine residue (Figure 4; Waas et al., 2005; Perche-Letuvee et al., 2014). The second step of wybutosine biosynthesis consists in the chemical transformation of *N-*methyguanosine (m^1^G_37_-tRNA) to 4-demethylwyosine (imG-14-tRNA). It is catalyzed by the TYW1 enzyme which belongs to the RS superfamily (Perche-Letuvee et al., 2012, 2014).

In the past 5 years, important insights into the reaction mechanism of TYW1 have been obtained. First, a longstanding question about the origin of the two carbon atoms required for the imidazoline ring formation was elucidated. Indeed, biochemical and labeling experiments proved that these two carbon atoms originate from the C_2_ and C_3_ of pyruvate and are incorporated into imG-14-tRNA with loss of C_1_ as formate or carbon dioxide (Young and Bandarian, 2011). Second, combined spectroscopic, biochemical and enzymological studies on chemically reconstituted *holo* TYW1 established that the enzyme contains two oxygen-sensitive [4Fe−4S]^2+/1+^ clusters, each ligated by only three cysteine residues that are absolutely required for activity (Perche-Letuvee et al., 2012). In contrast to MoaA, the RS cluster is located at the C-terminal half, it is ligated by the canonical CysX_3_CysX_2_Cys motif. The additional [4Fe-4S] cluster, located at the N-terminal part is coordinated by a CysX_12_CysX_12_Cys motif. Third, as expected, the site of hydrogen atom abstraction was identified to be the methyl group of m^1^G_37_-tRNA (Young and Bandarian, 2015). On the basis of these findings two mechanisms have been proposed (Perche-Letuvee et al., 2012; Young and Bandarian, 2015).

**Figure 5:**
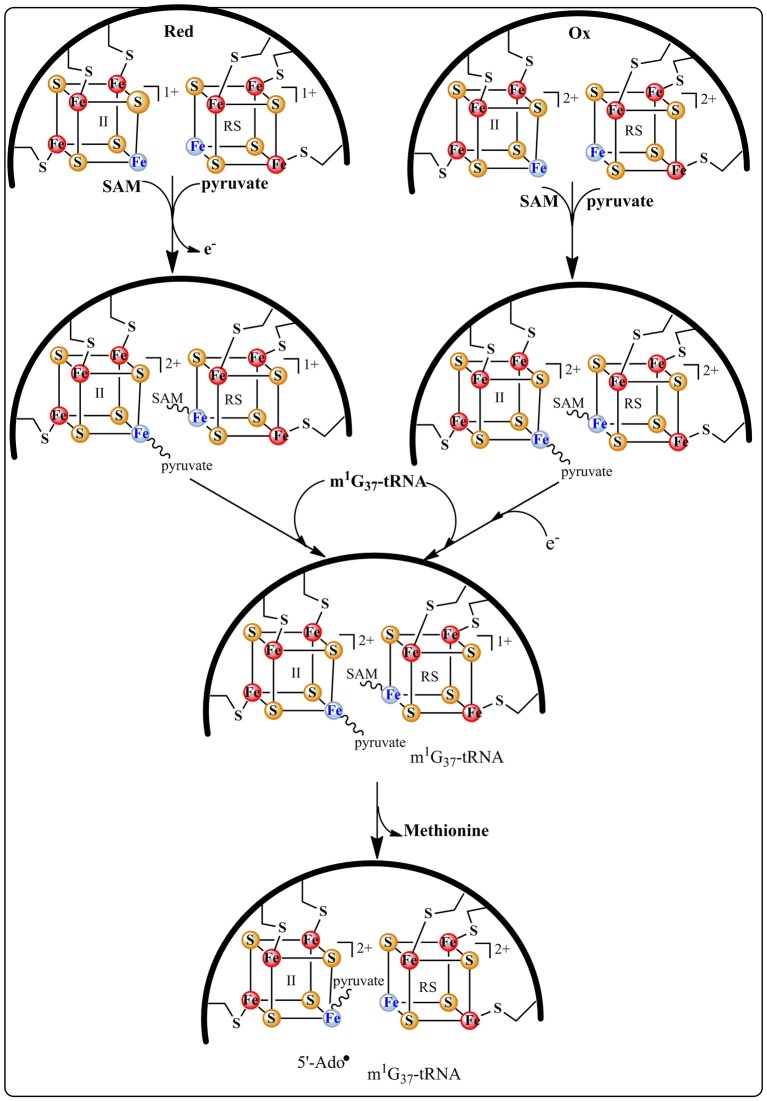
**Formation of the ternary complex *holo* TYW1, SAM and pyruvate**.

The group of Bandarian has proposed that the pyruvate co-substrate is activated via Schiff base formation with a conserved and essential lysine residue (Suzuki et al., 2007; Young and Bandarian, 2011). It was suggested that this Schiff base allows for the stabilization of the intermediates formed during catalysis (Young and Bandarian, 2011). However, no spectroscopic and biochemical data supporting this assumption are presently available. On the contrary, in our laboratory, biochemical and spectroscopic investigations demonstrated that TYW1 has evolved two different [4Fe-4S] clusters for the activation of the two co-substrates, SAM and pyruvate (Figure 5).

**Figure 6:**
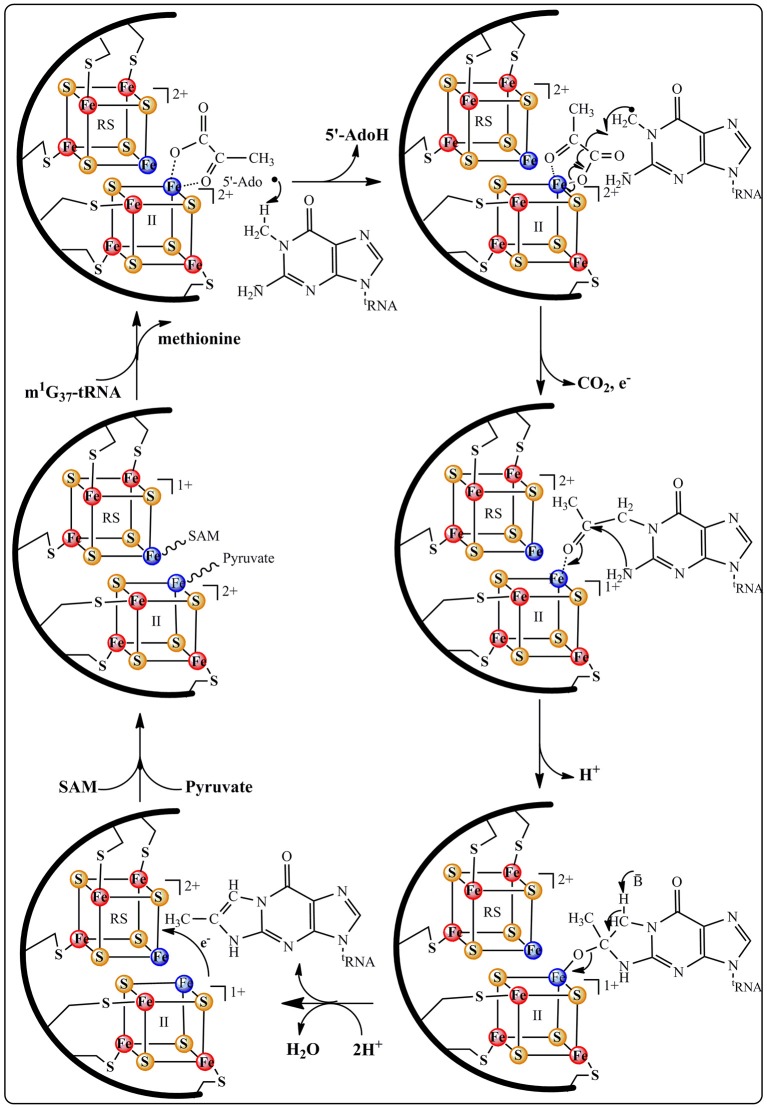
**Proposed catalytic mechanism for ImG_14_ biosynthesis catalyzed by TYW1 enzyme**.

This proposal is supported by EPR and Mössbauer data indicating that both co-substrates bind to the enzyme (Figure 5), SAM to the RS cluster whatever its oxidation state and pyruvate to the additional cluster in a way reminiscent of 5′-GTP binding to MoaA (see above). Moreover, these spectroscopic studies revealed that the addition of pyruvate to the reduced [4Fe-4S] additional cluster causes its re-oxidation with release of one electron (Perche-Letuvee et al., 2012). This unexpected oxidation is supposed to favor the polarization of the carbonyl group of pyruvate toward nucleophilic attack in the subsequent steps of biosynthesis (Figure 6). Once the ternary complex (*holo* TYW1, SAM, and pyruvate) is formed and only if the m^1^G_37_-tRNA substrate and electrons are present, SAM is cleaved to the 5′-Ado^•^ radical (Figure 5). The latter abstracts a hydrogen atom on the methyl group of m^1^G_37_-tRNA substrate with concomitant release of methionine and re-oxidation of the RS cluster at the 2+ state (Figure 6).

According to Figure 6, the m^1^G_37_-tRNA radical is proposed to add to carbon C_2_ of pyruvate still bound to the oxidized additional cluster leading to the homolytic cleavage of the C_1_–C_2_ bond and release of CO_2_ and one electron. The last two steps of the mechanism consist in the formation of the tricyclic ring system through nucleophilic addition of the 2-amino group of the base on the C_3_ of pyruvate followed by a general acid-base-catalyzed removal of H_2_O leading to ImG-14. This mechanistic proposal does not give the conserved lysine a direct role in the formation of the imidazoline. We propose that this lysine is important for the observed oxidation of the additional cluster upon interaction with pyruvate. Indeed, control of the redox state of [4Fe-4S] clusters by charged close residues has been recently documented in the case of the closely related RimO system (see below).

## MiaB and RimO enzymes: methylthiotransferases (MTTases)

**Figure 7:**
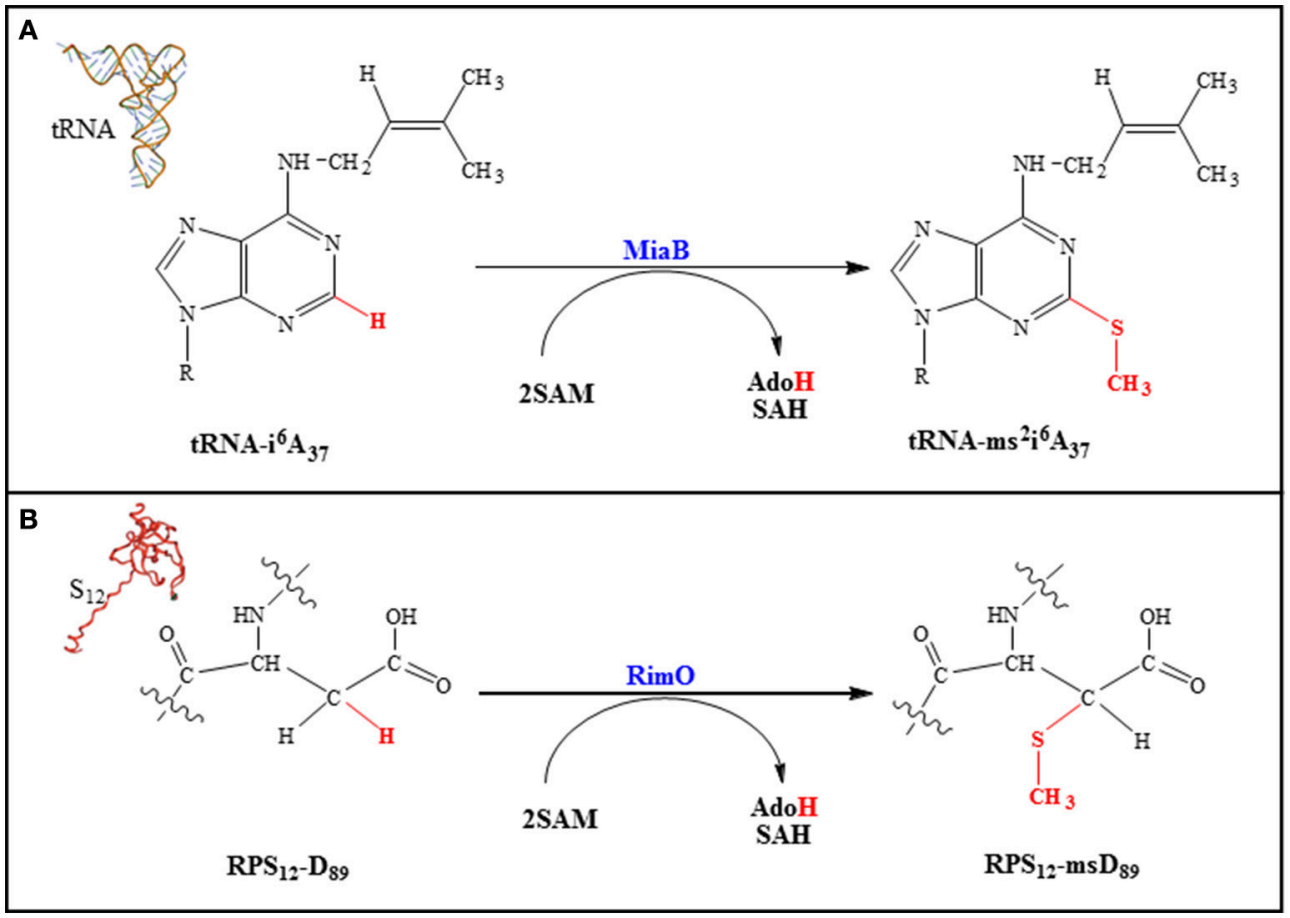
**MiaB (A)** and RimO **(B)**-catalyzed reactions.

According to phylogenetic analysis five families of MTTases have been identified (Arragain et al., 2010a; Atta et al., 2012) but only two have been biochemically, enzymatically and spectroscopically investigated (Atta et al., 2012). MiaB, represents the prototype of the first family. It catalyzes the transformation of N-6-isopentenyl adenosine (i^6^A_37_) into 2-methylthio-N-6-isopentenyl adenosine (ms^2^i^6^A_37_) in some tRNAs (Pierrel et al., 2002; Figure 7A). RimO belongs to the second family and is involved in the formation of a methylthiolated aspartate residue (RPS_12_ms-D_89_) in the ribosomal protein S12 (RPS_12_) (Anton et al., 2008; Figure 7B).

The function of the methylthio group in these macromolecules remains to be established even if some insight has been gained from the positioning of the modifications within the translation machinery (Gustilo et al., 2008). Indeed, a structural study reveals that the methylthio group at the 3′ nucleotide (A_37_) in the tRNA_Phe_ helps both to structure and optimize the codon/anticodon interaction (Jenner et al., 2010). Similarly, it has been shown that in RPS_12_ the methylthio group on D_89_ interacts with A_523_ of the 16S rRNA. This interaction allows for the structuration of a loop containing m^7^G_527_ in rRNA. Thus, the location of these modifications strongly suggests a role in codon–anticodon stability, translational efficiency and fidelity (Gustilo et al., 2008).

All MTTases comprise an N-terminal UPF0004 (Uncharacterized Protein Family 0004) domain of ~ 135 residues in length, which contains a CysX_35*-*36_CysX_32*-*33_Cys motif that binds the UPF [4Fe-4S] cluster. A central RS domain which is ~ 235 residues in length contains the CysX_3_CysX_2_Cys motif, and a C-terminal basic TRAM (TRM2 And MiaB) domain of ~ 60 residues in length which is typically found in tRNA-modifying enzymes (Atta et al., 2012). Note that RimO, which does not act on a nucleic acid substrate, also contains such a domain but with opposite polarity making it competent to bind the strongly basic RPS_12_ substrate (Arragain et al., 2010b). In order to achieve the methylthiolation reaction, all known MTTases so far display two different activities, both SAM dependent (Pierrel et al., 2004; Landgraf et al., 2013). Indeed, SAM is used as a methyl group donor in a SN_2_-based reaction and as a source of a 5′- Ado^•^ radical and both activities are expressed within a single polypeptide chain. Two classes of RS methyltransferases either depending on a cobalamin cofactor or not have recently been shown to face this very unusual situation. In GenK, a representative of the first class, one molecule of SAM methylates the cobalamin cofactor and a second one generates the 5′- Ado^•^ radical (Kim et al., 2013). For RlmN and Cfr, the representatives of the second class, which contain only a RS cluster, a first SAM molecule is used to methylate a cysteine residue close to the RS center and the second one to generate the 5′- Ado^•^ radical (Boal et al., 2011; Grove et al., 2011a,b). Based on insightful biochemical and spectroscopic studies of Cfr and RmlN and the X-ray structure of the latter, it was proposed that within its binding site SAM is ligand to the RS cluster and able to fulfill both functions (Grove et al., 2011b). However, there is no direct evidence for cysteine methylation being achieved by a SAM molecule coordinated to the cluster although the reaction is dependent on the presence of the cluster.

**Figure 8:**
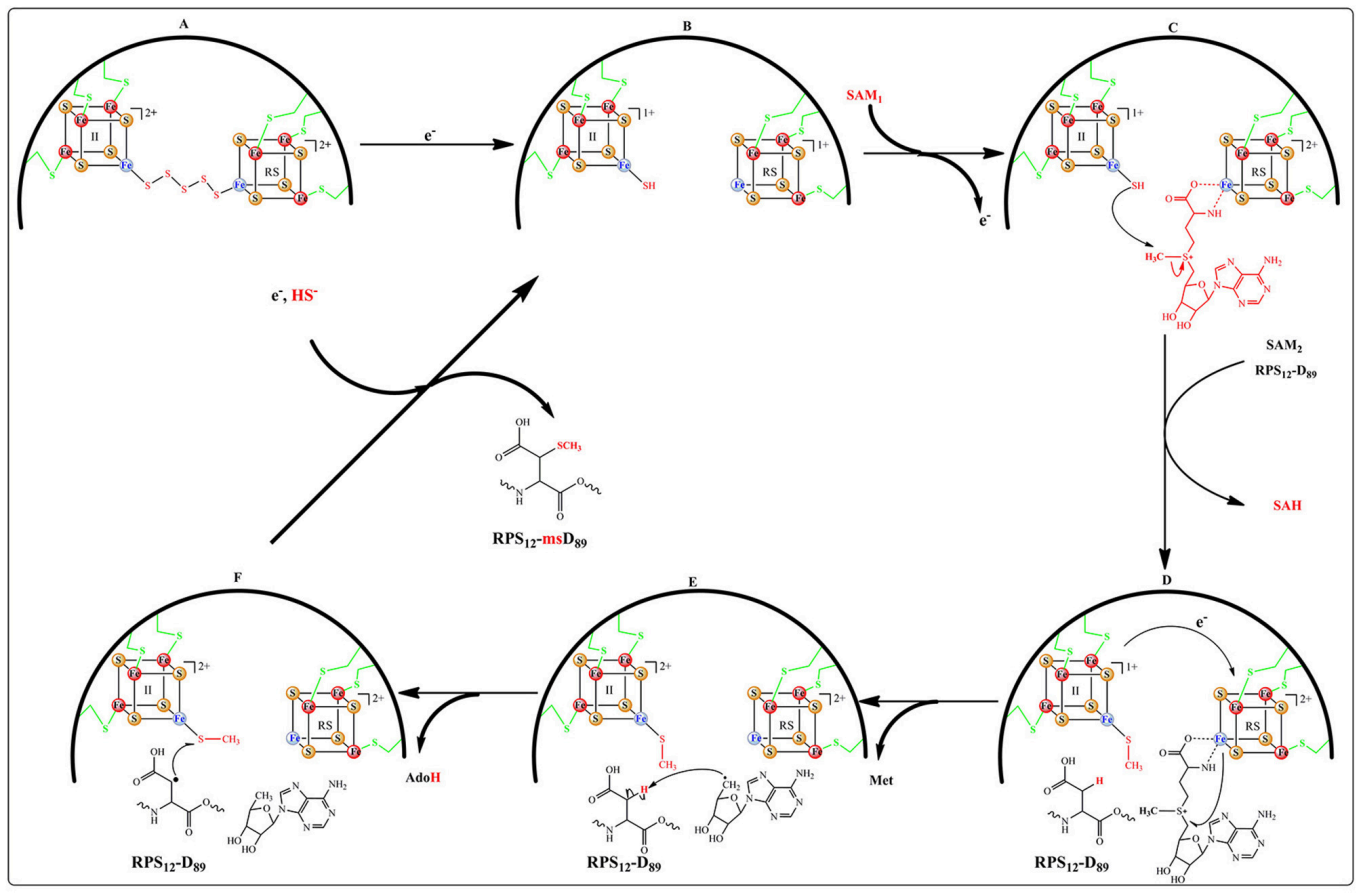
**Proposed catalytic mechanism for RPS_12_-msD_89_ biosynthesis catalyzed by RimO**.

Recent structural and biochemical *in vitro* studies done on MiaB and RimO showed that in the resting state (A) the two adjacent clusters are in the oxidized state and linked by a 8 Å spanning pentasulfide (Forouhar et al., 2013; Figure 8). In presence of electrons, both clusters are reduced presumably together with the pentasulfide leaving the unique iron of the UPF cluster with a hydrosulfide terminal ligand (state B). Coordination of a [4Fe-4S] cluster by three cysteines and a HS^−^ group has precedents in (*R*)-2-Hydroxyisocaproyl-CoA dehydratase (Knauer et al., 2011) and HydG involved in maturation of hydrogenases (Dinis et al., 2015). CW electrochemistry suggested that the clusters have very close redox potentials (−420 mV vs. NHE) in the range typical of other RS enzymes (Pierrel et al., 2003; Molle et al., 2016). Moreover, a detailed EPR and Mössbauer study of RimO conducted in the absence of substrate indicated that addition of SAM to the reduced protein triggers a very fast re-oxidation of the RS cluster and none detectable products resulting from the reductolysis of SAM (Molle et al., 2016). This event was accompanied on a longer time scale by the methylation of the hydrosulfide ligand bound to the UPF cluster (state C). An alternative mechanism has proposed that the pentasulfide is methylated on its ω position (Landgraf et al., 2013) but this seems unlikely considering the constrained active site revealed by the X-ray structure (Forouhar et al., 2013). Thus, in the case of RimO, it appears that two molecules of SAM with different functions bind successively to the RS cluster and that this dual activity is under redox control (Molle et al., 2016).

More recently, an electrochemical study of MiaB and RimO deposited as films on pyrolytic graphite electrode (PGE) confirmed the occurrence of a broad CV electrochemical wave (Maiocco et al., 2016). This signal could be fitted with two close one-electron signals at −390 and −450 mV vs. NHE. Based on a previous study of WT MiaB (Pierrel et al., 2003) these two signals were assigned to the UPF and RS cluster respectively (Maiocco et al., 2016). Upon SAM addition, square wave electrochemistry indicated a drastic shift of the low potential signal to around −650 mV suggesting the presence of a strongly stabilized oxidized cluster while the −390 mV signal remained. These data confirmed those obtained by the EPR and Mössbauer study mentioned above. However, in contrast to the conclusions of the latter study, the authors assigned the observed shift in potential to the UPF and not to the RS cluster and proposed that it resulted from the presence of a methyl sulfide ligand bound to the former although addition of methyl sulfide itself had a very weak effect on the electrochemical response (Maiocco et al., 2016). Interestingly, this study revealed that both enzymes were behaving alike but that the kinetic of the electrochemical response to SAM was much faster for RimO than for MiaB suggesting subtle differences between them.

For both enzymes the reductive cleavage of the second molecule of SAM to provide the canonical 5′-Ado^•^ radical (Figure 8, states D&E) depends on the presence of the substrate (i^6^A_37_-tRNA for MiaB and RPS_12_-D_89_ for RimO) (Arragain et al., 2010b; Forouhar et al., 2013). Furthermore, the X-ray structure of RimO shows that the two [4Fe-4S] clusters are ideally positioned (~ 8 Å) for one electron transfer from UPF to RS cluster (Figure 8, state E). Thus, from the available published data, MiaB and RimO mechanisms appear to have in common the four steps (A, B, C, and D) of Figure 8. However, they may differ regarding the activation of their substrate. Indeed, while in the case of RimO, H abstraction by 5′-Ado^•^ does not suffer from thermodynamic constraints (Figure 8 state E), in the case of MiaB, substrate activation requires abstraction of a hydrogen atom from the Adenine C_2_ sp^2^ carbon, generating a presumably energetically unfavorable σ-radical. This issue deserves to be investigated.

Recently, DFT calculations performed on RimO led us to propose two additional steps completing the catalytic cycle (Molle et al., 2016). First, they showed that the reaction takes place only when the UPF cluster is in the oxidized state. This suggests that binding of the substrate triggers an electron transfer from the UPF to the RS cluster, thereby making it competent in the cleavage of SAM_2_ (states D to E). Second, these calculations showed that the reaction proceeds through the attack of the substrate carbon radical on the methylthio co-substrate bound to UPF cluster (state F). Such a step has been proposed in the case of AlbA and related enzymes which catalyze intra peptidic C-S bond formation between cysteine and phenylalanine or threonine aminoacid residues of subtilosin A and other bacteriocins (Fluhe et al., 2012). Though, it is not yet established that this family of RS enzymes do contain coordinatively unsaturated additional clusters as described here. Regarding MTTases, several important questions related to the electron transfer steps remain to be addressed. In particular, it is important to understand where the electron removed from RS cluster goes to reach state C and how can this cluster accept an electron upon substrate interaction.

## LipA enzyme: lipoyl synthase

**Figure 9:**
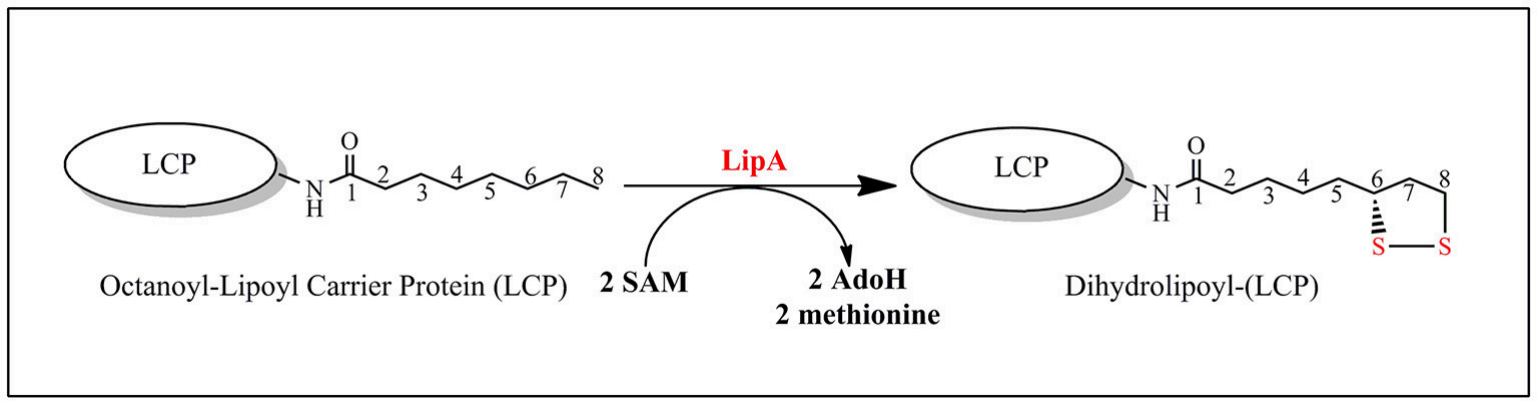
**LipA-catalyzed reaction**.

Lipoyl synthase (LipA) catalyzes the final step in the *de novo* biosynthesis of the lipoyl cofactor. The reaction consists in the insertion of two sulfur atoms at C_6_ and C_8_ of the octanoyl chain of metabolically critical 2-oxoacid dehydrogenases (Figure 9). However, the *in vitro* reaction is not catalytic as no more than 0.35 lipoyl group per LipA monomer can be obtained and this is probably explained by a lack of a suitable sulfur source in the assay. Biochemical and spectroscopic studies have shown that, in addition to the RS cluster, LipA contains and additional [4Fe-4S] cluster absolutely required for activity and that the reaction requires two SAM molecules, one per sulfur insertion (Cicchillo et al., 2004). The RS cluster is housed by the C-terminal half of the protein, while the additional cluster is bound to the N-terminal by a CysX_4_CysX_5_Cys motif.

**Figure 10:**
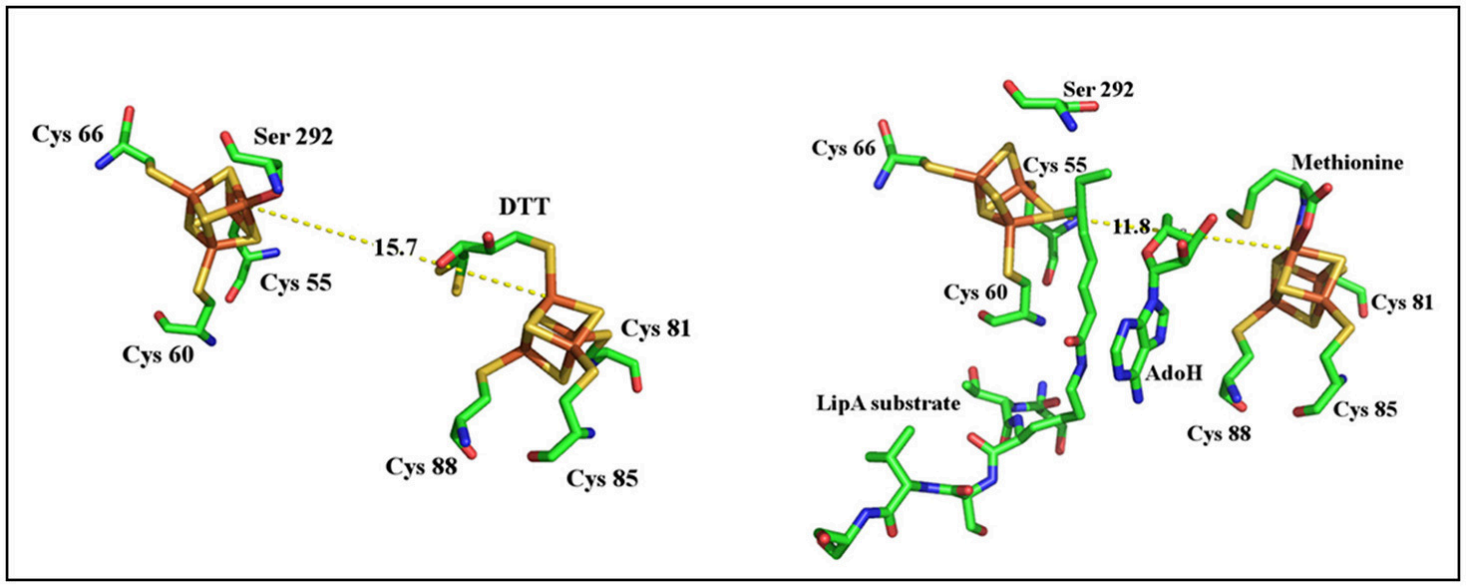
**Structural comparisons of substrate intermediate-bound (right)** and DTT-bound forms of LipA **(left)**.

In addition, the reaction is regiospecific at C_8_ and stereospecific with inversion at C_6_ (Parry and Trainor, 1978). Finally, studies with ^34^S-labeled protein suggest that both sulfur atoms are transferred from a single LipA molecule (Cicchillo and Booker, 2005). Recently, crystal structures of LipA from *Thermosynechococcus elongatus* (TeLipA) without substrate and from the human pathogen *Mycobacterium tuberculosis* both in the absence and presence of a substrate mimic have brought important insights on how the enzyme could manage the double sulfur insertion (Harmer et al., 2014; McLaughlin et al., 2016). The protein fold is typical of other RS enzymes and displays the usual α_6_β_6_ TIM barrel with appended extensions in the N-terminal and C-terminal regions. In the absence of substrate, the additional cluster is held by three conserved cysteines located in the N-terminal part of the sequence and secured by a strictly conserved serine residue belonging to a conserved motif at the very end of the C-terminal. The presence of a native serine ligand to an iron sulfur cluster is unprecedented and may serve for locking the enzyme in a conformation such as the two clusters are 15.4 Å apart (McLaughlin et al., 2016). Upon incubation with the substrate mimic, SAM and electrons an intermediate stage of the enzyme could be obtained and crystallized that revealed striking changes from the resting state. First, the serine ligand together with its subsite bound iron are expelled from the additional cluster allowing a drastic 4 Å move of the N-terminal bringing the two clusters 11.6 Å apart. In addition, a conserved arginine which, in the resting state, blocks the channel where the substrates bind is removed allowing for both the activation of the substrate by the RS cluster and its perfect positioning to accept a bridging sulfide from the additional cluster (Figure 10; McLaughlin et al., 2016).

**Figure 11:**
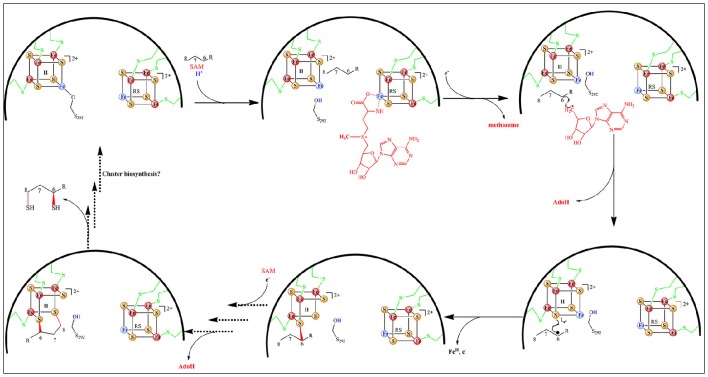
**Proposed mechanism for the lipoyl cofactor biosynthesis catalyzed by LipA**.

These events nicely match the data obtained with Mössbauer spectroscopy that revealed the formation of a [3Fe-4S] intermediate cluster during turnover (Lanz et al., 2015). The loss of the fourth iron is rationalized by the now ready access of the intermediate to one of the two accessible remaining bridging sulfides of the [3Fe-4S] cluster. Taking into account all of these data a reaction mechanism has been proposed (Figure 11) in which the fate of the additional cluster is to provide the sulfur atoms for the reaction. However, several important questions remain to be answered in order to validate this mechanistic proposal, the least one being not to address its relevance in the presence of a suitable exogenous sulfur source.

## Conclusions and perspectives

The realization that several RS enzymes such as MoaA and TYW1 described above contain an additional [4Fe-4S] displaying a free coordination site and involved in substrate (5′-GTP for MoaA) or co-substrate (pyruvate for TYW1) binding and activation lead us to propose and substantiate that the subsite iron in the additional cluster of MiaB and RimO could serve for the binding and delivery of a methylthio group during catalysis. Even if the utilization of [4Fe-4S] centers for substrate activation has well documented precedents as for example aconitase, co-substrate activation by coordinatively unsaturated FeS center as proposed here deserves to be considered. However, although the presence of an additional Fe-S cluster in the RS enzymes is historically connected to its discovery in BioB and LipA, the presently accepted mechanism of sulfur insertion brought about by the latter enzymes does not fit the one described in this review. Indeed, for these two enzymes, it is presently admitted that, *in vitro*, the additional cluster is sacrificed during the reaction in giving its bridging sulfides to the activated substrate (Booker et al., 2007; Jarrett, 2015) even if these systems proved to be catalytic *in vivo* (Choi-Rhee and Cronan, 2005). This sacrificial *in vitro* behavior is based on a large corpus of data but most if not all of them have been obtained in the absence of a suitable sulfur donor and the possibility remains that the observed reactions follow a dead-end pathway in which the substrate radical has no other alternative but to react with a bridging sulfide of the nearby additional cluster thereby destroying it. In agreement with this idea no repair system has still been proven to be efficient in sustaining turnovers in these enzymes and the very recent structural description of the LipA enzyme trapped in a bound half-sulfurated substrate intermediate strongly suggests that this structure does not represent the physiological dynamic one (McLaughlin et al., 2016). Thus, in this system, the nature of the sulfur donor remains to be clearly established.

In this review, we have shown that, when coordinatively unsaturated, the additional cluster of RS enzymes is used for binding and activating the enzyme substrate and/or co-substrate. It is important to realize that these functions appear to be extended in the case of HydG, one of the maturases of the Fe-Fe hydrogenase and NifB, a central player in the maturation of the FeMoco cofactor of nitrogenases. Recent spectroscopic and structural studies done on HydG indeed demonstrate that one iron of the additional cluster is linked to a dangler iron via a cysteine bridge (Dinis et al., 2015). This adduct is proposed to bind the dihydroglycine precursor of the CO and CN ligands ultimately found in the binuclear iron of the H cluster. In the case of NifB which contains, in addition to the RS cluster, not one but two additional [4Fe-4S] clusters (Wilcoxen et al., 2016), a recent advance has shown that, during the reaction, these additional clusters are fused into a 8Fe-9S-C structure (Hu and Ribbe, 2016). Strikingly, the central C_4_- carbide was shown to originate from the methyl group of SAM (Wiig et al., 2012). Moreover, when using SAM-CD_3_, volatile labeled methanethiol was produced during the reaction (Wiig et al., 2015). This led to the proposal that methanethiol derives from methylation of a bridging sulfur of one of the auxiliary [4Fe-4S] clusters. Considering that the NifB reaction balance involves the addition of one C and one S unit, an alternative mechanism may be envisioned, similar to the one recently established for RS MTTases, in which one of the additional [4Fe-4S] of NifB binds a hydrosulfide ligand amenable to methylation. The resulting CH_3_S ligand would then be processed to the final central carbide by successive one or two-electron oxidation reactions. However, it is not yet known if these additional clusters contain an iron site available for CH_3_SH coordination. These two examples show that additional clusters in RS enzymes may play unexpected and critical roles in the sophisticated chemistry at work in these enzymes. Many yet unsolved questions regarding these systems will continue to be a stimulating source of research on the fascinating role of auxiliary clusters present in Radical SAM enzymes.

## Author contributions

All authors listed, have made substantial, direct and intellectual contribution to the work, and approved it for publication.

### Conflict of interest statement

The authors declare that the research was conducted in the absence of any commercial or financial relationships that could be construed as a potential conflict of interest.
